# Effects of Vitrification on the Blastocyst Gene Expression Profile in a Porcine Model

**DOI:** 10.3390/ijms22031222

**Published:** 2021-01-27

**Authors:** Cristina Cuello, Cristina A. Martinez, Josep M. Cambra, Inmaculada Parrilla, Heriberto Rodriguez-Martinez, Maria A. Gil, Emilio A. Martinez

**Affiliations:** 1 Department of Medicine and Animal Surgery, Faculty of Veterinary Medicine, International Excellence Cam-pus for Higher Education and Research “Campus Mare Nostrum”, University of Murcia, 30100 Murcia, Spain; ccuello@um.es (C.C.); josepmiquel.cambra@um.es (J.M.C.); parrilla@um.es (I.P.); mariagil@um.es (M.A.G.); emilio@um.es (E.A.M.); 2 Institute for Biomedical Research of Murcia (IMIB-Arrixaca), Campus de Ciencias de la Salud, Carretera Bue-navista s/n, 30120 Murcia, Spain; 3 Department of Biomedical & Clinical Sciences (BKV), BKH/Obstetrics & Gynaecology, Faculty of Medicine and Health Sciences, Linköping University, SE-58185 Linköping, Sweden; heriberto.rodriguez-martinez@liu.se

**Keywords:** embryo, vitrification, transcriptome, blastocyst

## Abstract

This study was designed to investigate the impact of vitrification on the transcriptome profile of blastocysts using a porcine (*Sus scrofa*) model and a microarray approach. Blastocysts were collected from weaned sows (*n* = 13). A total of 60 blastocysts were vitrified (treatment group). After warming, vitrified embryos were cultured in vitro for 24 h. Non-vitrified blastocysts (*n* = 40) were used as controls. After the in vitro culture period, the embryo viability was morphologically assessed. A total of 30 viable embryos per group (three pools of 10 from 4 different donors each) were subjected to gene expression analysis. A fold change cut-off of ±1.5 and a restrictive threshold at *p*-value < 0.05 were used to distinguish differentially expressed genes (DEGs). The survival rates of vitrified/warmed blastocysts were similar to those of the control (nearly 100%, n.s.). A total of 205 (112 upregulated and 93 downregulated) were identified in the vitrified blastocysts compared to the control group. The vitrification/warming impact was moderate, and it was mainly related to the pathways of cell cycle, cellular senescence, gap junction, and signaling for TFGβ, p53, Fox, and MAPK. In conclusion, vitrification modified the transcriptome of in vivo-derived porcine blastocysts, resulting in minor gene expression changes.

## 1. Introduction

Since 1978, more than 7 million children have been born through Assisted Reproductive Technologies (ARTs) [1]. As the impact of ARTs increases, so do concerns about their potential health effects. The known risks of ARTs, including pregnancy complications, preterm birth, low birth weight, and highly increased birth defects, are relatively small [2]. Based on animal studies, it has been hypothesized that ART children may also have some long-term health consequences, such as higher risk of heart disease, diabetes, or hypertension [3]. Investigating the consequences of each ART on the embryos and their subsequent development may help us to create more efficient and safe procedures for patients. 

Because research on human embryos is limited by bioethics and legal issues, animal research in this area is essential [4]. The use of pigs as a model for translational research has unique advantages, since they have similar genetics to humans and a similar anatomy and physiology [5,6]. In this sense, porcine embryos have previously been used to investigate IVF-induced DNA methylation and gene expression disorders in blastocysts [7]. 

Among ARTs, the development and implementation of the vitrification of oocytes and embryos has been revolutionary in human-assisted reproduction, changing in vitro fertilization (IVF) practice [8], providing an increased efficiency of IVF treatment, improving pregnancy rates, and increasing the safety of assisted reproduction [9]. Today, vitrification is the gold standard worldwide for embryo cryopreservation in humans, but also in other mammalian species [10]. Several human studies based on metanalysis show that transfers performed with vitrified embryos reduce adverse perinatal effects related to severe ovarian stimulation syndrome [11]. However, in humans it is not possible to compare the outcomes of both kinds of embryos after transfer in a natural cycle, which may allow us to evaluate the impact of vitrification. In this sense, studies performed in pigs have described a higher pregnancy loss for vitrified embryos (10–20%) than that obtained after the transfer of fresh embryos (<2.5%) [12]. The mechanisms underlying this observation are unknown. Currently, we know that vitrification negatively impacts porcine embryo quality, augmenting levels of apoptosis [13,14] or impairing the ultrastructure [13]. Vitrification has been found to cause modifications in the embryo gene expression in several species [15,16,17,18,19,20]; however, the consequences of vitrification for the gene expression of mammalian embryos are still poorly known. Comparative studies on porcine are very scarce and limited to in vitro produced (IVP) embryos. 

Research based on RT-qPCR has demonstrated that vitrification significantly upregulates genes involved in both mitochondria and death receptor-mediated apoptotic pathways in porcine parthenogenic blastocysts [18]. It has also been reported that vitrification causes the downregulation of POU5F1, which is related to embryo implantation [17], and the upregulation of HSPA1A, which is involved in stress regulation [17]; vitrification also modifies the expression levels of IGF2- and IGF2R-imprinted genes [16] in IVP-produced porcine blastocysts. Although these studies are useful, they are focused on a few selected genes, and therefore the information about the vitrification impact on the embryo transcriptome is very limited. A better knowledge of the overall vitrification effects on the embryonic transcriptome may help us to understand sublethal cryoinjuries that could be associated with embryo developmental and pregnancy failure and would yield a new awareness about the response mechanisms of embryos undergoing vitrification. Such studies should be conducted on in vivo-derived porcine embryos to better discriminate the effects related exclusively to vitrification and warming. 

Microarrays or RNA-Seq analysis are the most efficient and comprehensive technologies that can provide a wide transcriptome coverage [21], enabling the assessment of the expression of thousands of genes with a single experiment. Therefore, this study used a microarray approach with RT-qPCR validation to investigate the effect of vitrification on the gene expression patterns of in vivo-derived embryos at the blastocyst stage using a pig (*sus scrofa*) model to determine if sub-lethal modifications, escaping the conventional morphology screening of embryo viability, are caused by the procedure.

## 2. Results

### 2.1. Embryo Collection and Embryo Viability

Blastocysts used in this study were collected from weaned sows (*n* = 13) at Day 6 (Day 0 = onset of estrus) of pregnancy. The mean ovulation rate of the donor sows was 20.8 ± 3.6 corpora lutea (range 14 to 25) and the recovery rate was 92.2%. Of the recovered structures, 96.0% were embryos and the rest were unfertilized oocytes and/or degenerated embryos. The total number of embryos collected was 239, of which 42.3%, 54.8%, and 2.9% were morulae, blastocysts, and hatched blastocysts, respectively. A total of 100 blastocysts were selected for use in this study, and the remaining embryos were used in other experiments. The survival rates of vitrified-warmed blastocysts (96.1 ± 3.4) were similar to those obtained in control embryos (100%).

### 2.2. Transcriptome Profiles of Vitrified-Warmed Blastocysts

**Figure 1:**
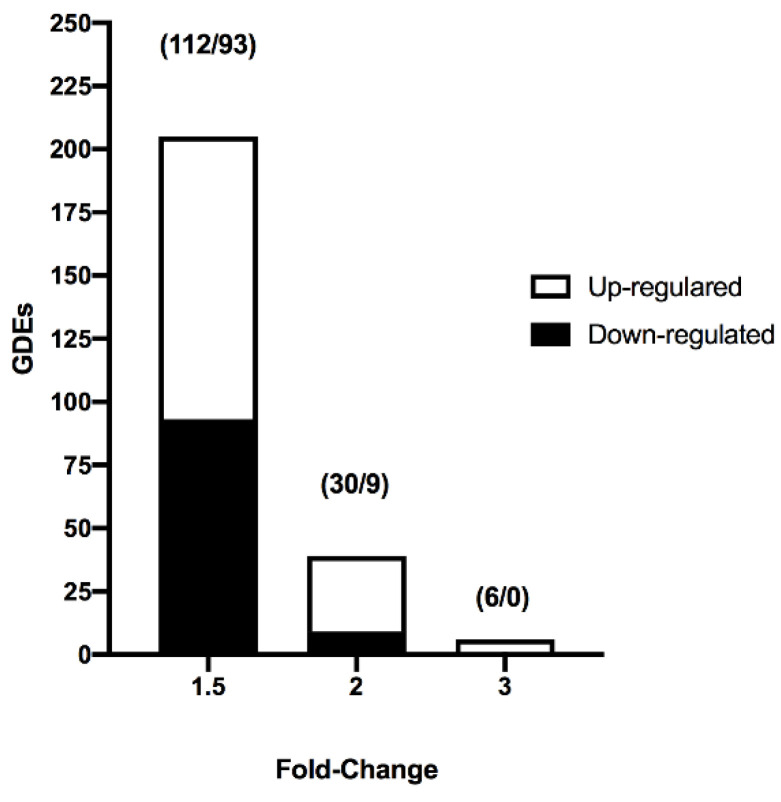
Number of differentially expressed genes (DEGs) identified from the transcriptome analysis of vitrified blastocysts using different fold change (1.5, 2, and 3) values and *p*-values < 0.05. Numbers within parentheses represent the number of upregulated and downregulated DEGs.

**Figure 2:**
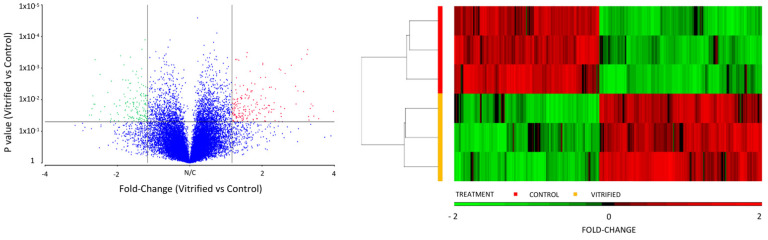
Volcano plot of differentially expressed genes (DEGs) between vitrified and control blastocysts. The significantly upregulated and downregulated genes at a log fold change cut-off of 1.5 and a *p*-value < 0.05 are represented with red and green dots, respectively, while unchanged genes are shown as blue dots. Unsupervised hierarchical clustering analysis of the DEGs of vitrified vs. control blastocysts. Columns and rows represent genes and samples, respectively. The color key under the heat map indicates the different expression levels.

We analyzed the effect of vitrification on the transcriptome profile of in vivo-derived porcine blastocysts. Vitrification and warming slightly affected the transcriptome profile of blastocysts. The PCA revealed that 68.8% of the variance was explained by the treatment (vitrification or not) of blastocysts. First, lists of the differentially expressed transcripts were generated using a restrictive threshold at *p*-value of 0.05 and various fold change cutoff values (Figure 1). Subsequent analyses were performed using the DEGs list produced from the selection criterion of fold change <−1.5 and >1.5. Using these parameters, 205 DEGs were identified in vitrified blastocysts compared to the control group. More specifically, 112 genes were upregulated (Supplementary Table S1), whereas 93 were downregulated (Supplementary Table S2). The DEGs detected in vitrified blastocysts are represented in the volcano plot (Figure 2). The unsupervised hierarchical clustering of transcriptome samples revealed that blastocyst vitrified samples could be clearly distinguished from the control samples (Figure 2).

### 2.3. Gene Ontology (GO) Enrichment Analysis of DEGs in Vitrified Blastocysts

**Figure 3:**
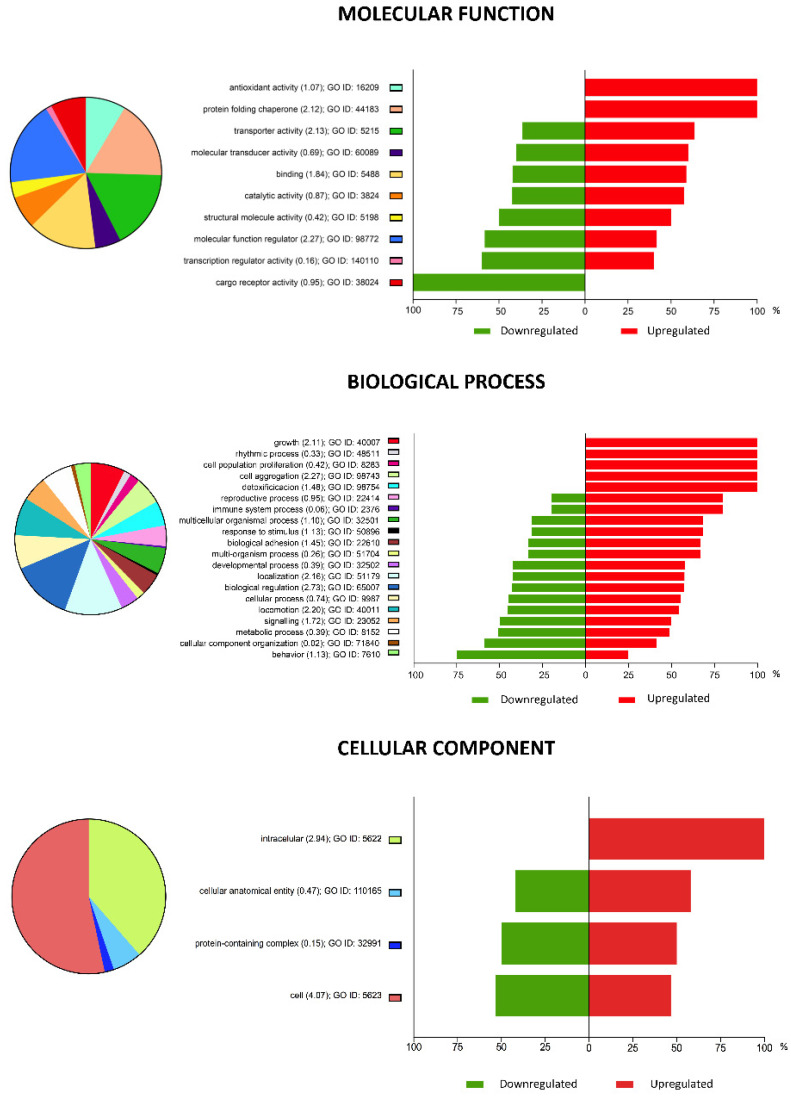
Pie chart representation of Gene Ontology (GO) terms for differentially expressed genes (DEGs) in vitrified blastocysts analyses, summarized according to molecular function, biological processes, and cellular component. The plots show the percentage of genes associated with each functional group that were upregulated (red) or downregulated (green). Functional categories of DEGs were obtained using GO annotations from the KEGG classification system. Numbers within parentheses indicate enrichment scores.

Gene Ontology (GO) analysis identified the main biological processes targeted by the DEGs. A total of 671 GO terms with an enrichment score ≥ 3 and an enrichment *p*-value < 0.05 were detected for vitrified blastocysts. Table 1 summarizes the top 10 most enriched GO terms corresponding to vitrified blastocysts. All the DEGs in vitrified blastocysts were classified within different functional categories based on their molecular function, biological process, and cellular component (Figure 3).

### 2.4. Kyoto Encyclopedia of Genes and Genomes (KEGG) Pathway Enrichment Analysis of DEGs in Vitrified Blastocysts

**Figure 4:**
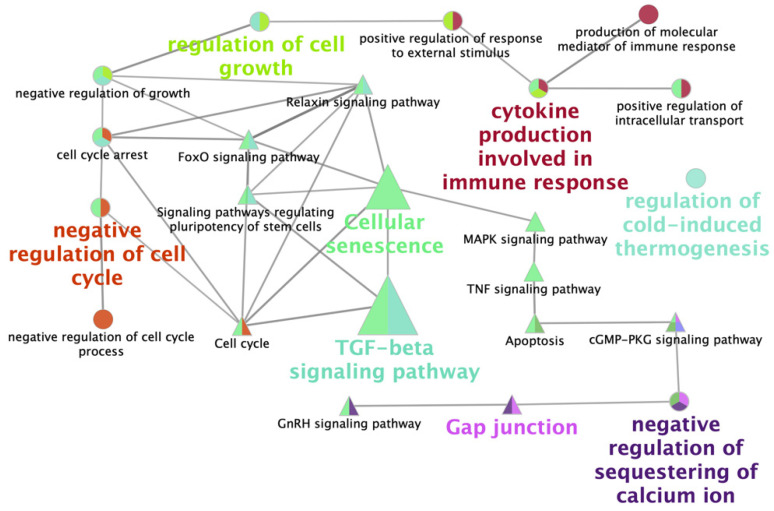
Functionally grouped network view of enriched pathways obtained from the analysis of differentially expressed genes in vitrified blastocysts compared to control embryos. The networks were obtained with the ClueGo v2.0.3 plug-in from the Cytoscape v3.0.0 software. Nodes are colored according to the grouping of related pathways, and groups are labelled according to the most significant pathway of the group. Edges indicate interactions between pathways and the node size represents the enrichment significance, where the largest nodes correspond to the highest significance.

Two gene lists, upregulated and downregulated, were analyzed to detect significant KEGG pathways. A total of ten enriched pathways for upregulated DEGs in vitrified blastocysts were detected (Table 2); the most enriched pathways in this group were the TGFβ, p53, and FoxO signaling pathways. Only four enriched pathways were obtained with downregulated DEGs (Table 3), including steroid biosynthesis, TGFβ, and cGMP-PKG signaling pathways and Gap junctions. A network of the main biological processes and pathways found when comparing the transcriptome profile of fresh and vitrified blastocysts analyzed with Cytoscape is represented in Figure 4. 

### 2.5. Validation of Microarray Results

**Figure 5:**
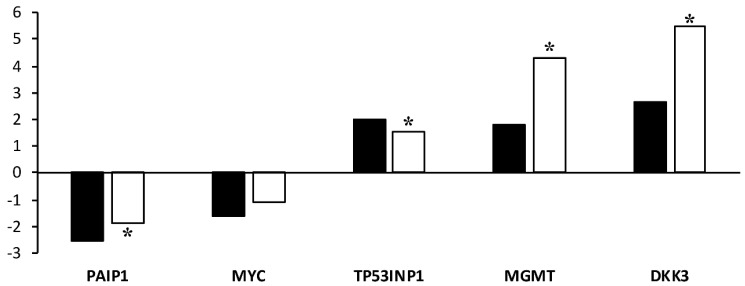
Validation of microarray results by RT-qPCR. The Y-axis represents the fold change between the vitrified and control blastocysts. * Asterisks indicate significant differences between the vitrified and control groups, as determined by RT-qPCR analysis.

The validation of the microarray data was performed by real-time quantitative PCR (RT-qPCR). The five genes validated by RT-qPCR showed an expression trend that was similar to the results observed in the microarrays (Figure 5). RT-qPCR analysis revealed that the mRNA levels for TP53INP, MGMT, and DKK3 were significantly (*p* < 0.05) upregulated. The expression of PAIP1 was significantly (*p* < 0.05) downregulated. The validation revealed that the expression of MYC was consistent with the results of the microarray, but the difference in expression levels between vitrified and control blastocysts analyzed by RT-qPCR was not significant. 

## 3. Discussion

To the best of our knowledge, this is the first report on the impact of vitrification/warming on the full transcriptome of in vivo-derived porcine blastocysts. This study contributes to the understanding of the consequences of vitrification/warming procedures on embryo quality and developmental competence, not only in pigs, but also in other mammalian species. Considering our results, the impact of vitrification was minor in terms of the number of DEGs. The impact of vitrification/warming was also moderate in terms of fold changes. We only found six DEGs in vitrified blastocysts showing a fold change greater than three. 

The GO term enrichment analysis of DEGs in vitrified blastocysts revealed that all the DEGs involved in the GO biological process of growth, cell population proliferation, cell aggregation and detoxification GO biological processes and those related to antioxidant activity and protein folding chaperone GO molecular functions were upregulated. These results reflect that the vitrification/warming process induced a stress-related response in blastocysts. Among the upregulated DEGs, the following genes included in these categories, the TP53INP (tumor protein p53 inducible nuclear protein 1) and CDKN1A (cyclin-dependent kinase inhibitor 1A (p21, Cip 1) genes, are of special interest for their role in the regulation of cell death and survival under stress conditions [22,23].

A KEGG pathway enrichment analysis of up- and downregulated genes of vitrified blastocysts showed that the impact of vitrification was moderate in terms of the number of pathways altered and the percentage of transcripts within each pathway that showed disturbed expression (range 0.7–11.1%). Although the disruption of gene expression can be considered minor, we should pay particular attention to modified pathways that are crucial for embryo development. 

Interestingly, when we examined the enriched pathways for upregulated genes in vitrified blastocysts, we observed important biological processes (cellular senescence and cell cycle) and signaling pathways (TGFβ, p53, and FoxO) that regulate the pluripotency of stem cells involved in embryo development and pregnancy. The enrichment of the TGFβ signaling pathway has also been reported in vitrified porcine COCs [24,25], and the upregulation of miRNAs related to this pathway was observed in vitrified mouse blastocysts [26]. The DKK3 gene, which was up regulated in vitrified blastocysts, is also involved in embryonic development and cell proliferation, this gene has been shown to be required for TGFβ-signaling during embryo development [27]. The upregulated genes in the TGFβ signaling pathway were BMPR1B, ID4, SMAD3 and TGFβ1, and there were also DEGs involved in the cell cycle pathway (CDKN1A) and signaling pathways regulating the pluripotency of stem cells (BMPR1B, ID4 and SMAD3). These genes are key regulators of cell proliferation, stem-cell state, differentiation, and apoptosis at the earliest stages of embryo development [28] and the TGFβ1 and SMAD3 are also key factors during embryo implantation [29,30]. The activation of the TGFβ-signaling pathway seems to be a response to cell stress and injury, as has been reported in epithelial cells [31]. The MYC, which belongs also to the TGFβ-signaling pathway, was repressed in vitrified blastocysts. The altered expression of this gene may have a negative effect disturbing the mechanism of cell competition in the blastocyst epiblast cells, which is regulated by the MYC levels [32]. The p53 signaling pathway is also an essential regulator of cellular stress, which may have opposite biological responses depending on many factors leading to cell death or cell survival [31]. The expression of TP53INP can be induced by many different stress signals [33] that have been described as consequences of embryo vitrification, such as oxidative stress [34,35] or DNA damage [13,36]. In conditions of reparable damage or transient stress, TP53INP induces cell cycle arrest by increasing the expression of CDKN1A/p21 [13,36], which was also upregulated in vitrified blastocysts in the present study. This response is related to repair, protection, and adaptation, and ultimately to cell survival [23]. Other important sing of the response of blastocysts to vitrification was the upregulation of MGMT. This important gene encodes a protein that is involved in DNA repair and in cellular defense against mutagenesis and toxicity agents [37]. The FoxO pathway is also activated under stress conditions [22,38]; FoxO molecules have been described in the inner cell mass of mouse blastocysts [39], and the upregulate target genes, thereby promoting cell cycle arrest genes in order to keep cells away from stress. If the cell cycle arrest is not sufficient to recover cells, apoptosis is activated, thus producing cell death [39]. In addition, the cellular senescence and cell cycle pathways, which play essential roles in cell response and cell repair under stressful conditions [39], were also enriched in the vitrified blastocysts. These results show the triggering of essential repair mechanisms in the vitrified blastocysts. 

Among the four enriched pathways obtained from the downregulated DEGs in vitrified blastocysts, the most remarkable is the steroid biosynthesis pathway. Historically, the production of steroids (estrogens) by porcine embryos has been considered the major signal for the maternal recognition of pregnancy [40]. Recently, it has been demonstrated that, although the production of embryonic estrogens is not essential for preimplantation development and early corpus luteum maintenance, it is indispensable for the maintenance of pregnancy beyond 30 days [41]. Therefore, the disruption of the steroid biosynthesis pathway could be, in part, responsible for the increased number of pregnancy failures that we have observed after transfer of vitrified blastocyst [12]. The enrichment of the Gap junction pathway that is involved in embryo-maternal interaction and implantation [41] could be also important. The HTR2B, which is a gene that belongs to this pathway, is involved in morphogenesis and development [42]; therefore, its repression could be detrimental for the embryo.

Comparing our results with previous results on the gene expression of vitrified porcine embryos performed by RT-qPCR, we observed very few matches due to the different origins of the embryos (parthenogenetic [43] or IVP [18]). Similar to Castillo-Martin et al. [16,17], we detected the upregulation of an HSP gene (HSPB1) in vitrified blastocysts; HSPB1 encodes a small heat shock protein involved in the response to environmental stress [44]. This result again shows the response of vitrified blastocysts to vitrification-induced stress. It is noteworthy to consider that these sub-lethal modifications caused by the procedure of vitrification/warming escaped our conventional morphological screening of embryo viability post-warming, which calls for a further screening of the extent of the transcriptomic alterations and their impact on embryo viability and for the development of more refined methods to discover these modifications at warming. 

## 4. Materials and Methods

### 4.1. Chemicals

Chemicals and media were acquired from Sigma-Aldrich Química S.A. (Madrid, Spain), unless otherwise indicated.

### 4.2. Animals

Embryos were obtained from hybrid donor sows (Landrace x Large-White) from the same genetic line (2 to 6 parities) located at a commercial farm (Agropor S.A., Murcia, Spain) and were maintained under field conditions, and placed individually in crates in a mechanically ventilated confinement facility. The sows were fed a commercial ration twice daily according to their nutritional requirements, with constant access to water.

### 4.3. Detection of Estrus and Artificial Insemination

Weaning was used to synchronize the estrus of the sows. Sows were evaluated for estrus once a day (at 7:00 a.m.), beginning the day after weaning. Sows were exposed to a vasectomized mature boar allowing snout-to-snout contact and considered in estrus when they showed a standing estrus reflex when applying manual back pressure. Only sows with an interval between weaning and estrus of 4 to 5 days were used as embryo donors. Estrus sows were artificially inseminated post-cervically at 6 and 24 h after the onset of estrus. Insemination doses (45 mL containing 1.5 × 109 spermatozoa) were prepared in a commercial artificial insemination center with ejaculates extended in Beltsville Thawing Solution extender (BTS; [45]. Sperm doses were stored at 17 °C for a maximum period of 24 h.

### 4.4. Embryo Recovery and Assessment

Embryos were surgically collected from the donor sows on Day 6 of the estrus cycle, with Day 0 being considered the onset of estrus. The sedation of embryo donors was performed with azaperone (Stresnil®, Landegger Strasse, Austria; 2 mg/kg body weight, i.m.) and general anesthesia was induced with sodium thiopental (B.Braun VetCare SA, Barcelona, Spain; 7 mg/kg body weight, i.v) and was maintained with 3–5% isoflurane gas (IsoFlo®, Madrid, Spain). The genital tract was exposed by performing a mid-ventral laparotomy. Then, the corpora lutea present in each ovary were counted and embryos were collected as previously described [46] by flushing the tip of each uterine horn with 30 mL of Tyrode’s lactate (TL)-HEPES- polyvinyl alcohol [47] with some modifications (TL-HESPES-PVA [48]. After flushing, the embryos collected from the uterine horn were evaluated under a stereomicroscope at a 60 x magnification, and the developmental stage and quality were assessed. One-cell structures and poorly developed embryos were classified as oocytes and degenerated embryos, respectively. Only unhatched full blastocysts with a good or excellent morphology according to the criteria of the International Embryo Transfer Society [49] and an intact zona pellucida were selected for the experiments. Collected embryos were washed three times in TL-HEPES-PVA, placed Eppendorf tubes containing 1.5 mL of this medium and transported to the University of Murcia (Murcia, Spain) in a transportable incubator set at 39 °C within 2 h after collection.

### 4.5. Vitrification and Warming

Vitrification and warming were performed according to a previously described protocol [50] in 4-well tissue culture plates (Nunc A/C, Roskilde, Denmark). The basic medium (BM) for vitrification and warming was TL-HEPES-PVA, and all media were held at 39 °C. Blastocysts were vitrified separately in groups of 4 to 6 embryos from a single donor within 4 h of embryo collection. Embryos were washed twice in BM at 39 °C and were subsequently equilibrated in BM containing 7.5% (*v/v*) dimethyl sulfoxide and 7.5% (*v/v*) ethylene glycol for 3 min; then, they were treated with BM supplemented with 16% (*v/v*) dimethyl sulfoxide, 16% (*v/v*) ethylene glycol and 0.4 M sucrose, for 1 min. After the last equilibration, embryos were located in a 1.5 μL drop and loaded into the narrow end of a superfine open pulled straw (SOPS; Minitüb, Tiefenbach, Germany) by capillary action. Immediately, the straw containing the embryos was plunged horizontally into liquid nitrogen. After one week of storage in liquid nitrogen, embryos were warmed by the one-step dilution method [51,52]. Briefly, the straw containing the embryos were removed from de liquid nitrogen and was immediately (less than one second) vertically submerged in a well containing 1mL of BM supplemented with 0.13 M sucrose and equilibrated in this medium for 5 min. Finally, embryos were washed in BM and then cultured in vitro for 24 h.

### 4.6. In Vitro Embryo Culture and Evaluation of In Vitro Embryo Viability Post-Warming

Post-warmed blastocysts were cultured in 4-well tissue culture plates; each well contained 500 μL per well of NCSU-23 [53] culture medium supplemented with 0.4 mg/mL of bovine serum albumin (BSA) and 10% (*v/v*) fetal calf serum, which was under paraffin oil (NidoilTM, Nidacon, Mölndal, Sweden) overlay. Embryo culture was performed in an incubator at 38.5 °C with 5% CO_2_ in air and 97% humidity atmosphere. After 24 h of in vitro culture, embryo morphology was assessed by stereomicroscopy to determine embryo viability and embryo developmental stage. The vitrified-warmed blastocysts that restructured their blastocoelic cavities after 24 h of in vitro culture and exhibited an excellent or good appearance were considered viable. Fresh control embryos that progressed after in vitro culture and showed good or excellent morphological features were classified as viable. The survival rate was calculated as the ratio of viable embryos to the total number of cultured embryos. Only viable embryos were selected for gene expression analysis. 

### 4.7. Sample Preparation and Microarray Hybridization

Total RNA was extracted from embryo samples (a pool of 10 embryos from different donors) was performed with a RNeasy Micro kit (P/N 74004; Qiagen Iberica, Madrid, Spain) according to manufacturer instructions. The isolated RNA was checked with a Nanodrop 2000 (ThermoFisher Scientific, Madrid, Spain) and a Bioanalyzer 2100 (Agilent, Santa Clara, CA, USA) to determine the total RNA amount and quality. The RNA integrity (RIN) values obtained ranged from 8 to 10. Then, ss-cDNA was synthesized from 650 pg of RNA from each sample using a GeneChip 3´ IVT Pico Reagent kit (P/N 902790; Affymetrix, ThermoFisher Scientific, Madrid, Spain), according to the protocol supplied by the manufacturer. The amount and quality of ds-cDNA was assessed by a Nanodrop 2000 (ThermoFisher Scientific) and a Bioanalyzer 2100 (Agilent, Santa Clara, CA, USA); ds-DNA targets were cleaned, and after fragmentation and terminal labelling, 4.5 µg of fragmented and biotinylated ds-DNA were added to a hybridization mix from a GeneChip Hybridization, Wash and Stain kit (P/N 90720; Affymetrix) according to the recommendations of the manufacturer. The resulting preparations were hybridized to the GeneChip® Porcine Genome Array (P/N 900624; Affymetrix), which assesses 20,201 genes, providing a widespread coverage of the *Sus scrofa* transcriptome. After scanning the array, the microarray data were processed using the Affymetrix Expression Command Console (Affymetrix), and all samples met the quality criteria. 

### 4.8. Microarray Data Analysis

The robust multiarray average (RMA) method was used to normalize the intensity data of each GeneChip® array [54], processing average intensity values according to the background adjustment. Raw values were then log2 transformed and quantile normalized in order to obtain a single intensity value for each probe set. Partek Genomics Suite and Partek Pathways software (Partek Incorporated, St. Louis, MS, USA) were used for the statistical analysis and biological interpretation of data. Principal component analysis (PCA) was used to provide the general configuration of the evaluated dataset and to observe variations in the transcriptome between samples. Statistical analysis was based on a single-factor ANOVA with a restrictive threshold at an un-adjusted *p*-value lower than 0.05 for selecting differentially expressed genes (DEGs). The analysis of the overrepresented Gene Ontology (GO) terms and pathways were analyzed based on the Kyoto Encyclopedia of Genes and Genomes (KEGG) database. Pathway networks were constructed using ClueGo v2.0.3 application from the Cytoscape v3.0.0 [55]. The ClueGo ontology source was the KEGG pathway database. In cytoscape, pathways were functionally grouped based kappa score (≥0.4). The following criteria were used for the ClueGo analysis: GO tree levels, 2–5 (first level = 0); minimum number of genes, 2; minimum percentage of genes, 2; GO term fusion; GO term grouping, initial group size of 2 and 50% for group merge. Data are expressed as means ± SD.

### 4.9. Quantitative Real-Time PCR (RT-qPCR) Analysis

For RT-qPCR, we analyzed total RNA from the same samples used for the microarrays that was reverse-transcribed to generate cDNA using a Maxima H Minus First Strand cDNA Synthesis Kit (Thermo Fisher Scientific, Waltham, Massachusetts, USA); conditions were 25 °C for 10 min, 50 °C for 15 min, and 85 °C for 5 min. Primers were designed using the Primer ExpressTM software v3.0.1 (Applied Biosystems, Foster City, CA, USA) and were commercially synthesized (primer sequences are shown in Table 4). 

The qPCRs were performed with the iTaqTM Universal SYBR Green Supermix in 10 μL volumes with 500 nM of each set of primers. All the reactions were carried out in a QuantStudioTM 5 Real-Time PCR System (Applied Biosystems, Waltham, MA, USA). The thermal cycling profile was 50 °C for 2 min for uracil-DNA glycosylase activation, 95 °C for 10 min for initial denaturation, followed by 40 cycles of 95 °C for 15 s and 60 °C for 1 min. A melt curve analysis was carried out to evaluate the specificity of each PCR by the detection of one single peak on the dissociation curve profile. A previous test with extra samples was conducted to calculate each primer pair efficiency according to the equation E = 10^(−1/slope)^. RT-qPCRs were run in triplicate per gene and per sample, and relative mRNA levels were quantified according to the Pfaffl method [56]. For data normalization, peptidylprolyl isomerase A (PPIA) was chosen as the housekeeping reference gene based on the results reported previously [57]. Gene efficiencies were calculated according to the equation E = 10^(–1/slope)^. The RT-qPCR data were analyzed by Student’s *t*-test using the IBM SPSS 24.0 Statistics package (IBM, Chicago, IL, USA). The normality of data was checked with the Shapiro–Wilk test and the distributions were parametric. The homogeneity of variance was determined using Levene’s test. A *p*-value of < 0.05 was considered statistically significant.

### 4.10. Experimental Design

To evaluate the effects of vitrification on the transcriptome of porcine in vivo-produced blastocysts, 13 weaned sows were used as embryo donors in three replicates. A total of 60 blastocysts were vitrified and warmed, and then they were cultured in vitro for 24 h. Control embryos were fresh blastocysts (*n* = 40) cultured in vitro for 24 h. After in vitro culture, the viability of vitrified-warmed and control embryos was assessed, and survival rates were calculated. Three different pools of 10 viable blastocysts each (from 4 different donors each pool) were prepared from each experimental group (vitrified and control). The three pools of vitrified and fresh blastocysts were similar because they were obtained from the same donors. Embryos were placed in 5 μL of phosphate-buffered saline (PBS) in RNAase free Eppendorf tubes and then stored at −80 °C until transcriptome analysis. A total of 5 genes (3 upregulated genes and 2 downregulated genes according to the microarray results; Table 4) were selected to confirm microarray results by RT-qPCR. For this validation, three biological replicates and three technical replicates per sample were assessed.

## 5. Conclusions

Taken together, our results demonstrate that vitrification modified the transcriptome of in vivo-derived porcine blastocysts, resulting in very minor gene expression changes. The changes in gene expression that we observed could were moderate in terms of the number of DEGs and fold change values. In vitrified blastocysts, we noted the activation of the cell cycle; cellular senescence; and the TFGβ, p53, FoxO, and MAPK signaling pathways in response to vitrification-induced stress. The disruption of pathways such as steroid biosynthesis and gap junctions could be related to the slight increased pregnancy loss observed after the transfer of vitrified embryos in a porcine model. Further research is needed to increase our knowledge of the biological implications of the GO terms and pathways modified by vitrification procedures.

## Figures and Tables

**Table 1:** Top ten most significant Gene Ontology (GO) terms for differentially expressed genes in vitrified blastocysts.

Biological Function	Type	Enrichment Score	Enrichment *p*-Value	% Genes in Group That are Present *
Protein kinase C-activating G protein-coupled receptor signalling pathway	BP	8.1	0.0003	37.5
ATPase regulator activity	MF	7.7	0.0004	33.3
Glucose transmembrane transport	BP	7.4	0.0006	30
Muscle cell cellular homeostasis	BP	7.1	0.0008	27.3
Hexose transmembrane transport	BP	7.1	0.0008	27.3
Positive regulation of smooth muscle cell chemotaxis	BP	6.9	0.0010	66.7
Monosaccharide transmembrane transport	BP	6.6	0.0014	23.1
Manganese ion transport	BP	6.3	0.0019	50
Manganese ion transmembrane transport	BP	6.3	0.0019	50
Hexose transmembrane transporter	MF	6.3	0.0019	50

BP: biological process. MF: molecular function. * % of genes in group that are present in the differentially expressed genes list (vitrified vs. control blastocysts).

**Table 2:** Enrichment analysis of pathways for upregulated genes in vitrified blastocysts.

Pathway Name	Pathway ID	Enrichment Score	Enrichment *p*-Value	Altered Genes (%) *	Gene List
TGFβ signaling pathway	kegg_pathway_161	6.7	0.0012	4.9	*BMPR1B, ID4, SMAD3, TGFB1*
p53 signaling pathway	kegg_pathway_133	5.1	0.0062	4.6	*CDKN1A, FAS, ZMAT3*
FoxO signaling pathway	kegg_pathway_126	5.0	0.0065	3.1	*CDKN1A, GABARAPL1, SMAD3, TGFB1*
Other types of O-glycan biosynthesis	kegg_pathway_44	4.9	0.0077	8.7	*B3GLCT, ST6GAL1*
Cellular senescence	kegg_pathway_154	4.4	0.0117	2.6	*CDKN1A, MAP2K6, SMAD3, TGFB1*
Glycerophospholipid metabolism	kegg_pathway_55	4.1	0.0173	3.2	*DGKA, MBOAT1, PLA2G16*
MAPK signaling pathway	kegg_pathway_115	3.7	0.0238	1.8	*DUSP6, FAS, HSPB1, MAP2K6, TGFB1*
Cell cycle	kegg_pathway_131	3.4	0.0324	2.5	*CDKN1A, SMAD3, TGFB1*
Signaling pathways regulating pluripotency	kegg_pathway_174	3.2	0.0420	2.2	*BMPR1B, ID4, SMAD3*
Glycerolipid metabolism	kegg_pathway_52	3.1	0.0455	3.4	*DGKA, MBOAT1*

Pathways were analyzed using the Pathways Kyoto Encyclopedia of Genes and Genomes (KEGG) database.* % of genes in pathway that are present in the differentially upregulated genes list (vitrified vs. control blastocysts).

**Table 3:** Enrichment analysis of pathways for downregulated genes in vitrified blastocysts.

Pathway Name	Pathway ID	Enrichment Score	Enrichment *p*-Value	Altered Genes (%) *	Gene List
Steroid biosynthesis	kegg_pathway_12	5.7	0.0035	10.5	*DHCR24, MSMO1*
TGF-beta signaling pathway	kegg_pathway_161	5.0	0.0066	3.7	*BAMBI, MYC, RBL1*
cGMP-PKG signaling pathway	kegg_pathway_120	5.0	0.0067	2.5	*AKT1, ITPR1, PLN, RGS2*
Gap junction	kegg_pathway_173	4.9	0.0073	3.5	*HTR2B, ITPR1, MAP3K2*

Pathways were analyzed using KEGG Pathways Kyoto Encyclopedia of Genes and Genomes (KEGG) database. * % of genes in pathway that are present in the differentially downregulated genes list (vitrified vs. control blastocysts).

**Table 4:** Sequences of the primers used for the quantitative real-time PCR (RT-qPCR) analysis.

Gene Symbol	Accession Number	Primer		Size	Efficiency
*PAIP1*	XM_003483818.4	Forward (5’-3’)	AATGCCCCTGAATTTTACCC	192	92.95
		Reverse (5’-3’)	ATCTGTTGTAACCCAGCCATTT		
*MYC*	NM_001005154.1	Forward (5’-3’)	TCGGACTCTCTGCTCTCCTC	157	102.05
		Reverse (5’-3’)	GCTGCCTCTTTTCCACAGAA		
*TP53INP1*	XM_001925224	Forward (5’-3’)	GCTGCCTCTTTTCCACAGAA	184	91.44
		Reverse (5’-3’)	TAAGATTTTGGCGACGAAGG		
*MGMT*	XM_005671579.3	Forward (5’-3’)	GGTCCAGAGGAGATGATGGA	208	94.23
		Reverse (5’-3’)	GGGCTGCTAACTGCTGGTAA		
*DKK3*	XM_005661123.3	Forward (5’-3’)	AAGACACGCAGCACAAACTG	163	84.56
		Reverse (5’-3’)	AAGACACGCAGCACAAACTG		
PPIA *	XM_021078519.1	Forward (5’-3’)	AGAAGTCTGAATGGGTTCCTCA	100	98.12
		Reverse (5’-3’)	CCAACCACTCAGTCTTGGCA		

* Housekeeping gene.

## Data Availability

The datasets used and/or analyzed during the current study are available from the corresponding author on reasonable request.

## Supplementary Materials

The following are available online at https://www.mdpi.com/1422-0067/22/3/1222/s1: Table S1: Differentially expressed transcripts in vitrified porcine blastocysts. Table S2: Differentially expressed downregulated genes in vitrified porcine blastocysts.
